# Development of a Rapid On-Site Method for the Detection of *Kazachstania servazzii* and *Candida sake* in Kimchi Using Loop-Mediated Isothermal Amplification

**DOI:** 10.4014/jmb.2502.02029

**Published:** 2025-04-23

**Authors:** Yoon-Soo Gwak, Gyeong-Eun Kim, Chan-Il Bae, Hae-Yeong Kim, Mi-Ju Kim

**Affiliations:** Institute of Life Sciences and Resources and Department of Food Science and Biotechnology, Kyung Hee University, Yongin 17104, Republic of Korea

**Keywords:** *Kazachstania servazzii*, *Candida sake*, kimchi, loop-mediated isothermal amplification, on-site detection method

## Abstract

Kimchi has recently gained considerable global interest for its health benefits, including its probiotic properties and anticancer and antioxidative effects. However, in the later stages of kimchi fermentation, an increase in the number of yeast species that form a white film on the surface of kimchi, such as *Kazachstania servazzii* and *Candida sake*, takes place, which can negatively affect kimchi quality. Thus, we developed loop-mediated isothermal amplification (LAMP) assays for the on-site detection of *K. servazzii* and *C. sake* in kimchi. The internal transcribed spacer (ITS) region of 24 yeast strains were aligned to identify the specific region of the target yeasts, after which the LAMP primer for each target species was designed. The developed LAMP primer sets specifically amplified *K. servazzii* and *C. sake* and detected 0.4 and 4 pg of *K. servazzii* and *C. sake* DNA, respectively. In addition, the limits of detection were 10^3^ CFU/ml for *K. servazzii* and 10^2^ CFU/ml for *C. sake*, respectively. The LAMP methods were validated by applying them to fermented kimchi samples, with the LAMP reactions being completed within 35 min. Overall, we found that the developed LAMP assays had excellent potential to be used for advanced on-site detection of *K. servazzii* and *C. sake* due to their specificity, sensitivity, and rapidity.

## Introduction

Kimchi is a traditional Korean fermented food prepared through the fermentation of vegetables such as cabbage, radish, and cucumber. It is then seasoned with various ingredients, including red pepper powder, green onion, garlic, ginger, and jeotgal (salted and fermented seafood) [1]. Currently, kimchi has gained considerable attention as a probiotic vegetable with several health benefits. Indeed, previous studies have confirmed that kimchi possesses probiotic properties, anticancer and antioxidative effects, and antiobesity and immune promotion functions and can alleviate allergies and atopic dermatitis [2, 3]. Furthermore, estimates have shown that the worldwide consumption of kimchi exceeds 1.5 million tons annually [4].

The fermentation process needed to create kimchi is naturally initiated by various microorganisms present in the raw ingredients, with approximately 200 species of bacteria and yeasts involved in this process [5, 6]. In the early stages of fermentation, less acid-tolerant lactic acid bacteria (LAB), such as *Leuconostoc citreum* and *Leuconostoc mesenteroides*, predominate. However, as fermentation proceeds and the kimchi becomes more acidic and less aerobic, *Lactiplantibacillus plantarum* and *Latilactobacillus sakei* become dominant [5]. In the later stages, the yeast population rapidly increases, marking the imminent completion of kimchi fermentation by LAB [7, 8]. At the late stages of fermentation, white colony forming yeast (WCFY) appears and forms white layers on the surface of the kimchi [8], which can easily cause consumer dissatisfaction and reduce the value of the kimchi product through film formation, softening texture, expansion of packages, and production of off-flavor and off-odor [8, 9]. *Kazachstania*, *Candida*, *Pichia*, *Debaromyces*, *Yarrowia*, *Hanseniaspora*, and *Rhodotorula* are the representative genera of WCFY [8, 10]. In particular, *Kazachstania servazzii* and *Candida sake* have been identified as the major WCFY species and require rapid detection during the monitoring process to prevent the deterioration of kimchi and economic losses [8, 11].

Although various detection methods have been used for targeting the nucleic acid of microbial species, loop-mediated isothermal amplification (LAMP) has emerged as a revolutionary molecular diagnostic technique that amplifies DNA under isothermal conditions [12]. This method uses two pairs of specifically designed inner and outer primers, in addition to two loop primers, to recognize six to eight distinct regions of the target species [12, 13]. This assay is rapid, specific, sensitive, and effective and requires only a portable device for on-site detection of the target [12, 14].

Therefore, the current study aimed to develop a rapid on-site detection method for the early identification of *K. servazzii* and *C. sake* using LAMP. We designed species-specific LAMP primers targeting a specific region of the internal transcribed spacer (ITS) of the two species. The specificity and sensitivity of the developed assays were then evaluated, and the methods were validated using the final product (*i.e.*, kimchi). Our results demonstrated that the developed method is capable of accurately and rapidly detecting *K. servazzii* and *C. sake*.

## Materials and Methods

### Yeast Strains

A total of 30 yeast strains, including *K. servazzii* and *C. sake*, were used to confirm the specificity of the developed LAMP assays (Table 1). Each strain was obtained from the Korean Collection for Type Cultures (Republic of Korea), Korean Agricultural Culture Collection (Republic of Korea), and the Korean Collection for Kimchi Microorganisms (Republic of Korea). Three yeast strains isolated from different food sources were also used. All yeast strains were cultured in YPD broth (MB Cell, Republic of Korea) at 25°C for 48 h.

### Kimchi Samples

Kimchi samples were produced using salted cabbage and seasoning at an 80:20 ratio. Kimchi was seasoned with red pepper powder, ground radish, garlic, ginger, green onion, fermented fish sauce, glutinous rice porridge, and water. Thereafter, the kimchi samples were fermented at 10°C for 50 days and subsequently used to validate the specificity and sensitivity of the LAMP assays for detecting *K. servazzii* and *C. sake*.

### DNA Extraction

All yeast strains were grown in two generations, with the final culture being used for DNA isolation. Kimchi samples were ground and filtered to prepare kimchi extract. Thereafter, 1 ml of yeast strains and kimchi samples were centrifuged, and the supernatant from each sample was discarded. The HiGene Genomic DNA Prep Kit For Microorganisms (Biofact, Republic of Korea) was used for DNA extraction following the manufacturer’s instructions with slight modifications. The purity and concentration of the isolated DNA were measured using a NanoReady Touch spectrophotometer (Life Real, China). DNAs with 260/280 nm values between 1.7 and 2.0 were used as the template DNA for specificity and sensitivity analyses, as well as the validation of the LAMP assays.

### Primer Design

Sequences of the ITS region for yeast species were obtained from National Center for Biotechnology Information GeneBank database. To identify the *K. servazzii*- and *C. sake*-specific regions, the sequences of 24 yeasts were aligned using the Clustal omega. The *K. servazzii*- and *C. sake*-specific LAMP primer sets were designed using Primer Explorer software version 5 (Eiken Chemical Co., Japan) and synthesized using Bionics (Republic of Korea). The sequences and information of the designed primers are summarized in Table 2.

### LAMP Reaction

LAMP reaction was performed in 25 μl of reaction volume. The LAMP mixture contained 15 μl of Isothermal Master Mix ISO-001 (OptiGene, UK), 0.2 μM of each outer primer (F3 and B3), 0.8 μM of each inner primer (FIP and BIP), 0.4 μM of each loop primer (LF and/or LB), and 40 ng of DNA template. A real-time fluorescence detector, Genie III LAMP detector (OptiGene), was utilized to perform the reaction. The isothermal amplification temperatures for the LAMP assays were 64 and 65°C for *K. servazzii* and *C. sake*, respectively. The amplification step lasted for 30 min, followed by the heating and cooling step from 98 to 80°C at a rate of 0.05°C/s for annealing curve analysis.

### Specificity and Sensitivity of LAMP Assays

The specificity of *K. servazzii*- and *C. sake*-specific LAMP primers was evaluated using genomic DNAs extracted from 30 yeast strains. The sensitivity tests for the *K. servazzii* and *C. sake* primer sets were performed with DNAs ranging from 40 ng to 0.4 pg. Furthermore, cultured broths of both species were used to verify the limit of detection (LOD) of the developed LAMP assays. The cell number was determined via the plate count method, and the cultured broths were serially diluted from 10^1^ to 10^6^ CFU/ml. Thereafter, diluted broths were used for DNA extraction.

### Validation of LAMP Assays

To validate the developed LAMP assay, naturally fermented kimchi samples and kimchi samples artificially inoculated with 2 × 10^6^ and 1.2×10^6^ CFU/g of *K. servazzii* and *C. sake* were used. All kimchi samples were packaged in 25 g and were fermented at 10°C for 50 days. Kimchi samples were obtained at 10-day intervals to determine changes in the yeast as kimchi fermentation progressed.

## Results and Discussion

### Development of Species-Specific LAMP Primer

**Fig. 1:**
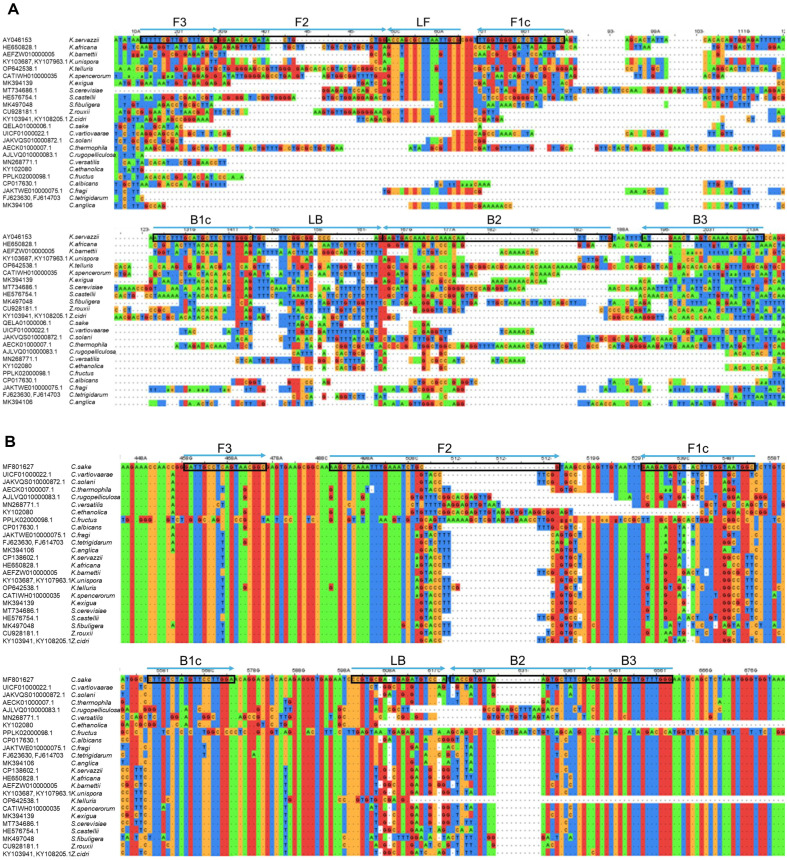
Sequence alignment of (A) *Kazachstania servazzii*- and (B) *Candida sake*-specific LAMP primers in the internal transcribed spacer (ITS) region in comparison to other yeast species.

In the current study, we developed LAMP assays to detect *K. servazzii* and *C. sake*. To design species-specific LAMP primer sets, information regarding the ITS regions of 24 yeast strains were downloaded from the GeneBank database. Thereafter, the sequences for all yeast strains were aligned using Clustal omega, which enabled the identification of a specific region for the target species. Subsequently, *K. servazzii*- and *C. sake*-specific LAMP primer sets were designed based on Accession No. AY046153 and MF801627, respectively. The binding sites for the LAMP primer sets are presented in Fig. 1, and detailed information about the primer sets is provided in Table 2. Given that the LAMP assay uses two pairs of inner and outer primers, two loop primers can be used additionally [12, 13]. The *K. servazzii*-specific LAMP primer designed herein consisted of two outer primers, two inner primers, and two loop primers binding to eight regions of the target gene. Meanwhile, the primer set for *C. sake* consists of two outer primers, two inner primers, and a backward loop primer binding to seven regions of target gene (Fig. 1 and Table 2). Thus, these LAMP assays can show higher specificity compared to conventional PCR or real-time PCR, which amplify only two or three sites of the target gene.

### Specificity and Sensitivity Results of the LAMP Assays for *K. servazzii* and *C. sake*

The specificity of the LAMP primer sets was evaluated using DNAs isolated from 30 yeast strains, including the target yeast species *K. servazzii* and *C. sake*. Our specificity results showed that the species-specific primer sets detected only the target species without cross-reactivity. Moreover, annealing curve analysis confirmed no amplification results in non-target species (Table 1). Furthermore, the yeast strains did not produce false negative results (Table 1). The aforementioned results clearly indicate that *K. servazzii*- and *C. sake*-specific LAMP primer sets have high specificity.

**Fig. 2:**
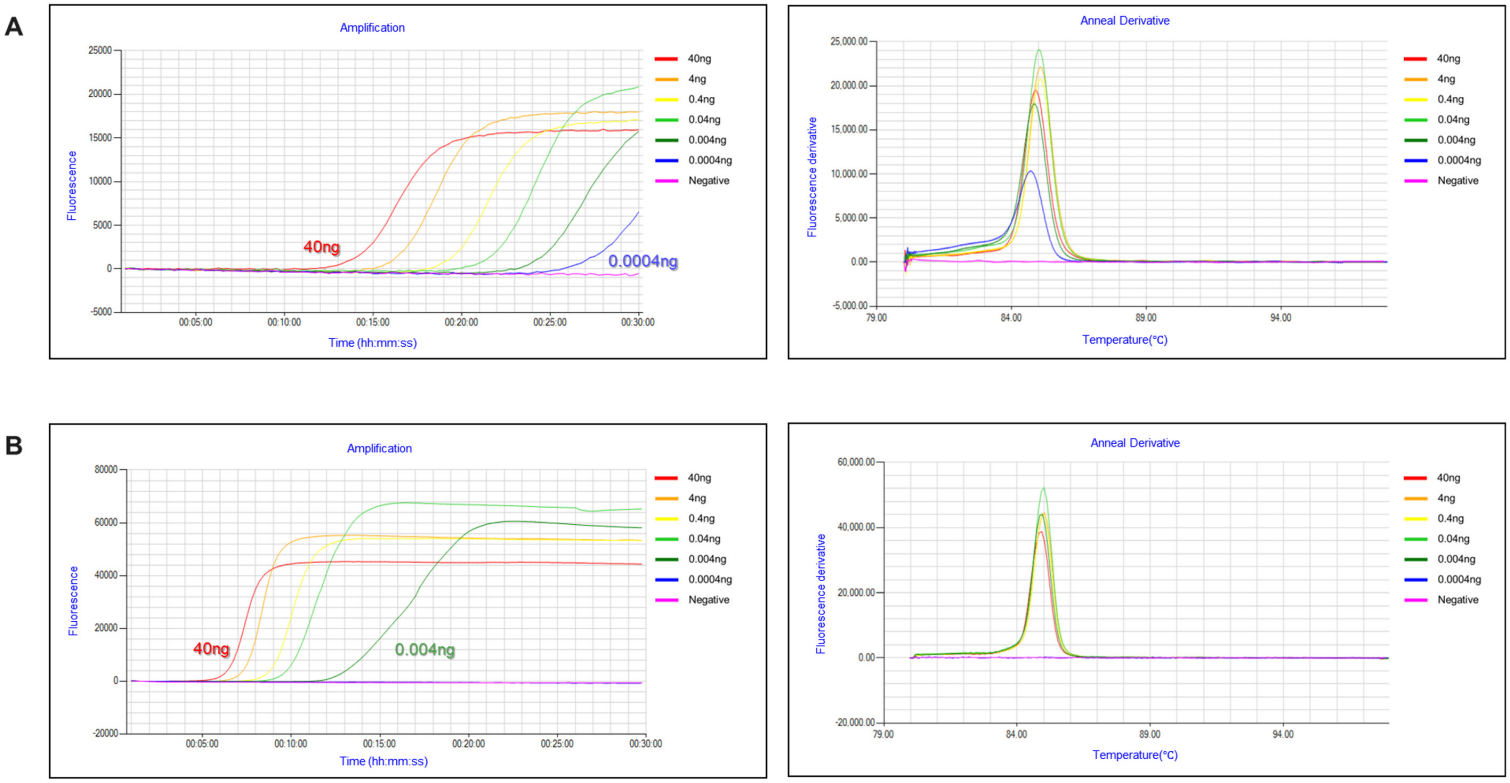
Sensitivity results of the developed LAMP assays for (A) *Kazachstania servazzii* and (B) *Candida sake*.

The sensitivity of each LAMP assay was determined using 10-fold serially diluted *K. servazzii* and *C. sake* DNAs ranging from 40 ng to 0.4 pg. Notably, we found that the *K. servazzii*- and *C. sake*-specific LAMP assays demonstrated positive signals up to 0.4 pg of *K. servazzii* DNA and 4 pg of *C. sake* DNA (Fig. 2), indicating that the developed LAMP methods had sufficient sensitivity for detecting *K. servazzii* and *C. sake*.

The LOD of each LAMP assay was also evaluated using DNAs isolated from various concentrations of *K. servazzii* and *C. sake* ranging from 10^6^ to 10^1^ CFU/ml. To assess reproducibility, the LOD test was performed in triplicate on different days. Accordingly, our findings revealed that the *K. servazzii*-specific LAMP assay showed positive signals in all three LOD tests at a concentration of 10^3^ CFU/ml but in only one reaction at a concentration of 10^2^ CFU/ml (Table 3). In contrast, the *C. sake*-specific LAMP assay detected 10^2^ CFU/ml of target species in all three LOD test (Table 3). To the best of our knowledge, there are no previous studies using the LAMP method for yeast detection, and the LODs of these assays are difficult to compare with those of other LAMP methods. However, a previous study showed that undesirable white colonies were observed on the surface of kimchi when the yeast number increased to 6 log CFU/g [10], suggesting a sufficient LOD for monitoring *K. servazzii* and *C. sake* in kimchi samples.

### Validation of LAMP Assays Using Kimchi

To determine the effects of the kimchi matrix on the LAMP assays, the developed LAMP assays were applied to real kimchi samples. As such, *K. servazzii* and *C. sake* were artificially inoculated into kimchi samples at a concentration of 2 × 10^6^ and 1.2 × 10^6^ CFU/g, respectively, and fermented at 10°C for 50 days. Additionally, kimchi samples fermented naturally under the same condition with no inoculation were used as the control sample. DNA from each sample was extracted at 10-days intervals and used as a template for the validation test.

*K. servazzii*- and *C. sake*-specific LAMP assays were used to identify the presence of target yeasts during the 50 days of fermentation. As shown in Table 4, all control kimchi samples from days 0 to 50 contained both yeasts. Kimchi samples inoculated with *K. servazzii* and *C. sake* were also used to validate the LAMP assay, with our results showing that all inoculated kimchi samples contained the corresponding yeast species (Table 4). These results indicate that the developed LAMP assays can effectively identify undesirable yeast species that affect the quality of kimchi.

Previous study [15] developed the real-time PCR assay to quantify *K. servazzii* that causes the spoilage of packaged food due to its gas production. However, real-time PCR takes 1–2 h to complete the reaction, whereas LAMP assays require only 35 min to detect target species [16]. Furthermore, LAMP assays require no sophisticated devices and only use a portable real-time fluorescence detector for LAMP reaction. Thus, the developed LAMP assays have excellent potential for application as a point-of-care detection method for *K. servazzii* and *C. sake*, which may have negative effects in foods.

## Conclusion

Our findings showed that the developed LAMP assays could not only detect *K. servazzii* and *C. sake* within 35 min but also do so with high specificity and sensitivity. Furthermore, the developed methods can be successfully applied to kimchi. To date, no studies have developed LAMP methods for the detection of *K. servazzii* and *C. sake* and applied it to actual kimchi samples. Therefore, the methods developed in the current study will certainly prove advantageous in the food industry by enabling the identification of yeast species that promote deterioration in product quality and facilitating the application of enhanced quality control techniques.

## Figures and Tables

**Table 1:** Specificity results of the LAMP assays for *Kazachstania servazzii* and *Candida sake*.

No.	Strain	Yeast species	LAMP results (annealing temperature, °C)	
			*K. servazzii*-specific LAMP	*C.sake*-specific LAMP
1	KCTC27365	*Kazachstania servazzii*	+^a^ (85.1 ± 0.1 ^b^)	−
2	KCKM0660	*Kazachstania servazzii*	+^a^ (85.4 ± 0.2 ^b^)	−
3	KCTC7654	*Candida sake*	−	+^a^ (84.9 ± 0.1 ^b^)
4	KCKM0659	*Candida sake*	−	+^a^ (84.8 ± 0.1 ^b^)
5	KCTC17479	*Kazachstania africana*	−	−
6	KCTC17222	*Kazachstania barnettii*	−	−
7	KCTC7169	*Kazachstania unispora*	−	−
8	KCTC7253	*Kazachstania telluris*	−	−
9	KCTC17227	*Kazachstania spencerorum*	−	−
10	KCTC17660	*Kazachstania exigua*	−	−
11	KACC48234	*Saccharomyces cerevisiae*	−	−
12	KACC48329	*Saccharomyces cerevisiae*	−	−
13	KACC30008	*Saccharomyces cerevisiae*	−	−
14	KCTC27604	*Saccharomyces castellii*	−	−
15	KCTC17621	*Saccharomyces cerevisiae* *var. cerevisiae*	−	−
16	KACC46781	*Saccharomyces fibuligera*	−	−
17	KACC30001	*Zygosaccharomyces rouxii*	−	−
18	KCTC7675	*Zygosaccharomyces cidri*	−	−
19	KCTC27005	*Candida vartiovaarae*	−	−
20	KCTC7689	*Candida solani*	−	−
21	KCTC17233	*Candida thermophila*	−	−
22	KCTC7629	*Candida rugopelliculosa*	−	−
23	KCTC17260	*Candida versatilis*	−	−
24	KCTC17366	*Candida ethanolica*	−	−
25	KCTC17392	*Candida fructus*	−	−
26	KCTC7122	*Candida albicans*	−	−
27	KCTC17643	*Candida fragi*	−	−
28	KFOY0028	*Candida tetrigidarum*	−	−
29	KFOY0030	*Candida tetrigidarum*	−	−
30	KFOY0062	*Candida anglica*	−	−

^a^Positive and negative results were shown as ‘+’ and ‘−’, respectively.

^b^Mean ± SD obtained from triplicate reactions.

**Table 2:** Sequences of LAMP primer sets used in this study.

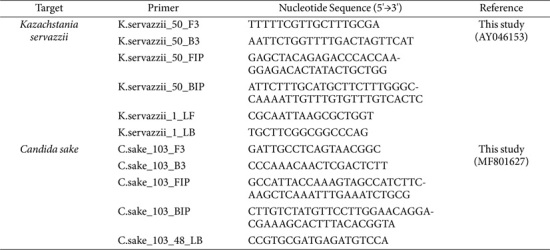

**Table 3:** LOD results of the LAMP assays for *Kazachstania servazzii* and *Candida sake*.

Concentration (CFU/ml)	LAMP results	
	*K. servazzii*-specific LAMP	*C. sake*-specific LAMP
10^6^	+ + + ^a^ (85.5 ± 0.4 ^b^)	+ + + (84.1 ± 0.3)
10^5^	+ + + (85.2 ± 0.7)	+ + + (84.2 ± 0.3)
10^4^	+ + + (84.9 ± 0.7)	+ + + (84.3 ± 0.3)
10^3^	+ + + (85.2 ± 0.3)	+ + + (84.4 ± 0.0)
10^2^	+ – – (84.8)	+ + + (84.2 ± 0.2)
10^1^	– – –	– – –

^a^Positive and negative results were shown as ‘+’ and ‘−’, respectively.

^b^Mean ± SD obtained from triplicate reactions.

**Table 4:** LAMP results in naturally fermented kimchi samples and spiked kimchi samples.

Fermentation time (day)	*Kazachstania servazzii*-specific LAMP		*Candida sake*-specific LAMP	
	Control kimchi sample	*K. servazzii*-inoculated kimchi sample	Control kimchi sample	*C. sake*-inoculated kimchi sample
0	+^a^	+	+	+
10	+	+	+	+
20	+	+	+	+
30	+	+	+	+
40	+	+	+	+
50	+	+	+	+

^a^Positive and negative results were shown as ‘+’ and ‘−’, respectively.
